# Supplementary material for: Effects of a 3-Week Hospital-Controlled Very-Low-Calorie Diet in Severely Obese Patients
Source: Nutrients. 2021 Dec 14;13(12):4468. doi: 10.3390/nu13124468 (PMC8703721; doi:10.3390/nu13124468)
Supplement: Supplementary file 1 [file nutrients-13-04468-s001.zip › nutrients-1477140-supplementary.pdf]

CONFIDENTIAL

STUDY NUMBER: ☐☐☐☐☐☐☐☐

FOOD FREQUENCY QUESTIONNAIRE

This questionnaire asks for some background information about you, especially about what you eat.

Your answers will be treated as strictly confidential and will be used only for medical research.

PLEASE COMPLETE USING BLACK INK/PEN

Date of birth: ☐☐ ☐☐ ☐☐☐☐

Please enter: **M if you are Male**
F if you are Female ☐

Please answer every question. If you are uncertain about how to answer a question then do the best you can, but please do not leave a question blank.

CAMB/PQ/6/1205

1. **YOUR DIET LAST YEAR**

For each food there is an amount shown, either a “medium serving” or a common household unit such as a slice or teaspoon. Please put a tick (✓) in the box to indicate how often, **on average**, you have eaten the specified amount of each food **during the past year**.

EXAMPLES:

EXAMPLE

For white bread the amount is one slice, so if you ate 4 or 5 slices a day, you should put a tick in the column headed “4-5 per day”.

FOODS AND AMOUNTS	AVERAGE USE LAST YEAR								
**BREAD AND SAVOURY BISCUITS** (one slice or biscuit)	Never or less than once/month	1-3 per month	Once a week	2-4 per week	5-6 per week	Once a day	2-3 per day	4-5 per day	6+ per day
White bread and rolls								✓	

For chips, the amount is a “medium serving”, so if you had a helping of chips twice a week you should put a tick in the column headed “2-4 per week”.

FOODS AND AMOUNTS	AVERAGE USE LAST YEAR								
**POTATOES, RICE AND PASTA** (medium serving)	Never or less than once/month	1-3 per month	Once a week	2-4 per week	5-6 per week	Once a day	2-3 per day	4-5 per day	6+ per day
Chips				✓					

For very seasonal fruits such as strawberries and raspberries you should estimate your average use when the fruits are in season, so if you ate strawberries or raspberries about once a week when they were in season you should put a tick in the column headed “once a week”

FOODS AND AMOUNTS	AVERAGE USE LAST YEAR								
**FRUIT** (1 fruit or medium serving)	Never or less than once/month	1-3 per month	Once a week	2-4 per week	5-6 per week	Once a day	2-3 per day	4-5 per day	6+ per day
Strawberries, raspberries, kiwi fruit			✓						

Please estimate your average food use as best you can, and please answer every question – do not leave ANY lines blank. PLEASE PUT A TICK (✓) ON EVERY LINE

FOODS AND AMOUNTS	AVERAGE USE LAST YEAR								
**MEAT AND FISH** (medium serving)	Never or less than once/month	1-3 per month	Once a week	2-4 per week	5-6 per week	Once a day	2-3 per day	4-5 per day	6+ per day
Beef: roast, steak, mince, stew or casserole									
Beefburgers									
Pork: roast, chops, stew or slices									
Lamb: roast, chops or stew									
Chicken or other poultry eg. turkey									
Bacon									
Ham									
Corned beef, Spam, luncheon meats									
Sausages									
Savoury pies, eg. meat pie, pork pie, pasties, steak & kidney pie, sausage rolls									
Liver, liver paté, liver sausage									
Fried fish in batter, as in fish and chips									
Fish fingers, fish cakes									
Other white fish, fresh or frozen, eg. cod, haddock, plaice, sole, halibut									
Oily fish, fresh or canned, eg. mackerel, kippers, tuna, salmon, sardines, herring									
Shellfish, eg. crab, prawns, mussels									
Fish roe, taramasalata									
	Never or less than once/month	1-3 per month	Once a week	2-4 per week	5-6 per week	Once a day	2-3 per day	4-5 per day	6+ per day

Please check that you have a tick (✓) on EVERY line

PLEASE PUT A TICK (✓) ON EVERY LINE

FOODS AND AMOUNTS	AVERAGE USE LAST YEAR								
**BREAD AND SAVOURY BISCUITS** (one slice or biscuit)	Never or less than once/month	1-3 per month	Once a week	2-4 per week	5-6 per week	Once a day	2-3 per day	4-5 per day	6+ per day
White bread and rolls									
Brown bread and rolls									
Wholemeal bread and rolls									
Cream crackers, cheese biscuits									
Crispbread, eg. Ryvita									
**CEREALS** (one bowl)									
Porridge, Readybrek									
Breakfast cereal such as cornflakes, muesli etc.									
**POTATOES, RICE AND PASTA** (medium serving)									
Boiled, mashed, instant or jacket potatoes									
Chips									
Roast potatoes									
Potato salad									
White rice									
Brown rice									
White or green pasta, eg. spaghetti, macaroni, noodles									
Wholemeal pasta									
Lasagne, moussaka									
Pizza									
	Never or less than once/month	1-3 per month	Once a week	2-4 per week	5-6 per week	Once a day	2-3 per day	4-5 per day	6+ per day

Please check that you have a tick (✓) on EVERY line

PLEASE PUT A TICK (✓) ON EVERY LINE

FOODS AND AMOUNTS	AVERAGE USE LAST YEAR								
**DAIRY PRODUCTS AND FATS**	Never or less than once/month	1-3 per month	Once a week	2-4 per week	5-6 per week	Once a day	2-3 per day	4-5 per day	6+ per day
Single or sour cream (tablespoon)									
Double or clotted cream (tablespoon)									
Low fat yogurt, fromage frais (125g carton)									
Full fat or Greek yogurt (125g carton)									
Dairy desserts (125g carton)									
Cheese, eg. Cheddar, Brie, Edam (medium serving)									
Cottage cheese, low fat soft cheese (medium serving)									
Eggs as boiled, fried, scrambled, etc. (one)									
Quiche (medium serving)									
Low calorie, low fat salad cream(tablespoon)									
Salad cream, mayonnaise (tablespoon)									
French dressing (tablespoon)									
Other salad dressing (tablespoon)									
**The following on bread or vegetables**									
Butter (teaspoon)									
Block or hard margarine, eg. Stork, Krona (teaspoon)									
Polyunsaturated margarine, eg. Flora, sunflower, soya spreads (teaspoon)									
Soft margarines,including olive oil based and dairy spreads, eg. Blue Band, Clover (teaspoon)									
Low fat spreads (less than 60% fat), eg. Outline, Gold (teaspoon)									
Very low fat spread (less than 30% fat) (teaspoon)									
	Never or less than once/month	1-3 per month	Once a week	2-4 per week	5-6 per week	Once a day	2-3 per day	4-5 per day	6+ per day

Please check that you have a tick (✓) on EVERY line

PLEASE PUT A TICK (✓) ON EVERY LINE

FOODS AND AMOUNTS	AVERAGE USE LAST YEAR								
**SWEETS AND SNACKS** (medium serving)	Never or less than once/month	1-3 per month	Once a week	2-4 per week	5-6 per week	Once a day	2-3 per day	4-5 per day	6+ per day
Sweet biscuits, chocolate , eg. digestive (one)									
Sweet biscuits, plain, eg. Nice, ginger (one)									
Cakes eg. fruit, sponge, home baked									
Cakes eg. fruit, sponge, ready made									
Buns, pastries eg. scones, flapjacks, home baked									
Buns, pastries eg. croissants, doughnuts, ready made									
Fruit pies, tarts, crumbles, home baked									
Fruit pies, tarts, crumbles, ready made									
Sponge puddings, home baked									
Sponge puddings, ready made									
Milk puddings, eg. rice, custard, trifle									
Ice cream, choc ices									
Chocolates, single or squares									
Chocolate snack bars eg. Mars, Crunchie									
Sweets, toffees, mints									
Sugar added to tea, coffee, cereal (teaspoon)									
Crisps or other packet snacks, eg. Wotsits									
Peanuts or other nuts									
**SOUPS, SAUCES, AND SPREADS**									
Vegetable soups (bowl)									
Meat soups (bowl)									
Sauces, eg. white sauce, cheese sauce, gravy (tablespoon)									
Tomato ketchup (tablespoon)									
Pickles, chutney (tablespoon)									
Marmite, Bovril (teaspoon)									
Jam, marmalade, honey (teaspoon)									
Peanut butter (teaspoon)									
	Never or less than once/month	1-3 per month	Once a week	2-4 per week	5-6 per week	Once a day	2-3 per day	4-5 per day	6+ per day

Please check that you have a tick (✓) on EVERY line

PLEASE PUT A TICK (✓) ON EVERY LINE

FOODS AND AMOUNTS	AVERAGE USE LAST YEAR								
**DRINKS**	Never or less than once/month	1-3 per month	Once a week	2-4 per week	5-6 per week	Once a day	2-3 per day	4-5 per day	6+ per day
Tea (cup)									
Coffee, instant or ground (cup)									
Coffee, decaffeinated (cup)									
Coffee whitener, eg. Coffee-mate (teaspoon)									
Cocoa, hot chocolate (cup)									
Horlicks, Ovaltine (cup)									
Wine (glass)									
Beer, lager or cider (half pint)									
Port, sherry, vermouth, liqueurs (glass)									
Spirits, eg. gin, brandy, whisky, vodka (single)									
Low calorie or diet fizzy soft drinks (glass)									
Fizzy soft drinks, eg. Coca cola, lemonade (glass)									
Pure fruit juice (100%) eg. orange, apple juice (glass)									
Fruit squash or cordial (glass)									
**FRUIT** **For seasonal fruits marked *, please estimate your average use when the fruit is in season**									
Apples (1 fruit)									
Pears (1 fruit)									
Oranges, satsumas, mandarins (1 fruit)									
Grapefruit (half)									
Bananas (1 fruit)									
Grapes (medium serving)									
Melon (1 slice)									
* Peaches, plums, apricots (1 fruit)									
* Strawberries, raspberries, kiwi fruit (medium serving)									
Tinned fruit (medium serving)									
Dried fruit, eg. raisins, prunes (medium serving)									
	Never or less than once/month	1-3 per month	Once a week	2-4 per week	5-6 per week	Once a day	2-3 per day	4-5 per day	6+ per day

Please check that you have a tick (✓) on EVERY line

PLEASE PUT A TICK (✓) ON EVERY LINE

FOODS AND AMOUNTS	AVERAGE USE LAST YEAR								
**VEGETABLES** Fresh, frozen or tinned (medium serving)	Never or less than once/month	1-3 per month	Once a week	2-4 per week	5-6 per week	Once a day	2-3 per day	4-5 per day	6+ per day
Carrots									
Spinach									
Broccoli, spring greens, kale									
Brussels sprouts									
Cabbage									
Peas									
Green beans, broad beans, runner beans									
Marrow, courgettes									
Cauliflower									
Parsnips, turnips, swedes									
Leeks									
Onions									
Garlic									
Mushrooms									
Sweet peppers									
Beansprouts									
Green salad, lettuce, cucumber, celery									
Watercress									
Tomatoes									
Sweetcorn									
Beetroot									
Coleslaw									
Avocado									
Baked beans									
Dried lentils, beans, peas									
Tofu , soya meat, TVP, Vegeburger									
	Never or less than once/month	1-3 per month	Once a week	2-4 per week	5-6 per week	Once a day	2-3 per day	4-5 per day	6+ per day

Please check that you have a tick (✓) on EVERY line

2. Are there any **OTHER** foods which you ate more than once a week? Yes ☐ No ☐

If **YES,** please list below

Food	Usual serving size	Number of times eaten each week

3. What type of milk did you most often use?

Select one only

- Full cream/whole ☐
- Skimmed ☐
- Dried milk ☐
- Other, specify ☐
- Semi-skimmed ☐
- Channel Islands, gold ☐
- Soya ☐
- None ☐

4. How much milk did you drink each day, including milk with tea, coffee, cereals etc?

- None ☐
- Quarter of a pint ☐
- Half a pint ☐
- Three quarters of a pint ☐
- One pint ☐
- More than one pint ☐

5. Did you usually eat breakfast cereal (excluding porridge and Ready Brek mentioned earlier)?

Yes ☐ No ☐

If **YES,** which brand and type of breakfast cereal, including muesli, did you usually eat?

List the one or two types most often used

Brand e.g. Kellogg's	Type e.g. cornflakes

6. What kind of fat did you most often use for frying, roasting, grilling etc?

Select one only

- Butter ☐
- Lard/dripping ☐
- Vegetable oil ☐
- Solid vegetable fat ☐
- Margarine ☐
- None ☐

If you used vegetable oil, please give type eg. corn, sunflower ☐

7. What kind of fat did you most often use for baking cakes etc?

Select one only

- Butter ☐
- Lard/dripping ☐
- Vegetable oil ☐
- Solid vegetable fat ☐
- Margarine ☐
- None ☐

If you used margarine, please give name or type eg. Flora, Stork ☐

8. How often did you eat food that was fried at home?

Daily ☐ 1-3 times a week ☐ 4-6 times a week ☐
Less than once a week ☐ Never ☐

9. How often did you eat fried food away from home?

Daily ☐ 1-3 times a week ☐ 4-6 times a week ☐
Less than once a week ☐ Never ☐

10. What did you do with the visible fat on your meat?

Ate most of the fat ☐ Ate as little as possible ☐
Ate some of the fat ☐ Did not eat meat ☐

11. How often did you eat grilled or roast meat? ☐☐ times a week

12. How well cooked did you usually have grilled or roast meat?

Well done /dark brown ☐ Lightly cooked/rare ☐
Medium ☐ Did not eat meat ☐

13. How often did you add salt to food while cooking?

Always ☐ Rarely ☐
Usually ☐ Never ☐
Sometimes ☐

14. How often did you add salt to any food at the table?

Always ☐ Rarely ☐
Usually ☐ Never ☐
Sometimes ☐

15. Did you regularly use a salt substitute (eg LoSalt)? Yes ☐ No ☐

If **YES**, which brand? ______________________

16. During the course of last year, on average, how many times a week did you eat the following foods?

**Food type**	**Times/week**	**Portion size**
Vegetables (not including potatoes)	☐☐	medium serving
Salads	☐☐	medium serving
Fruit and fruit products (not including fruit juice)	☐☐	medium serving or 1 fruit
Fish and fish products	☐☐	medium serving
Meat, meat products and meat dishes (including bacon, ham and chicken)	☐☐	medium serving

17. Have you taken any vitamins, minerals, fish oils, fibre or other food supplements during the past year?

- ☐ Yes
- ☐ No
- ☐ Sometimes
- ☐ Don't know

If **YES or SOMETIMES,** please complete the table below.

If you have taken more than 8 types of supplement please put the most frequently consumed brands first.

Example: If you take one tablet of vitamin C two times a day, please write '2' in the amount-column and tick (✓) the 'once a day' box. Most supplements mention a strength value (in our example 500mg), please write this information in the table.

Supplements				Average frequency for the past year<br>Tick (✓) **ONE** box per line to show how often on average you took the amount consumed as mentioned in 'amount' column.					
Brand	Name	Strength (strength of the supple-ment for each tablet or capsule)	Amount (number of tablets, capsules or teaspoons taken in one day)	Never or less than once a month	1-3 per month	Once a week	2-4 per week	5-6 per week	Once a day
Boots	High strength vitamin C	500mg	2 tablets						✓

EXAMPLE

Thank you for your help

Prehrambene namirnice i količne **Prosječan unos namirnice unutar godine dana**

Meso i ribe (srednje velika porcija)	Nikad ili < od 1 mjesečno	1-3 puta mjesečno	1 tjedno	2-4 puta tjedno	5-6 puta tjedno	1 dnevno	2-3 puta dnevno	4-5 puta dnevno	6 ili > puta dnevno
Govedina: pečena, odrezak, mljevena, (120g.) gulaš (330g.)									
Goveđi hamburger									
Svinjetina: pečena, kotlet, (120g.) gulaš (330g.)									
Janjetina: pečena, kotlet (120g.), gulaš (330g.)									
Piletina i druga perad (130 g.)									
Slanina (40 g.)									
Šunka (40 g.)									
Konzervirano meso: usoljena govedina, mesni doručak (40 g.)									
Kobasice (45 g.)									
Mesne pite, bureci od mesa, kobasica u tijestu (160 g.)									
Jetrica, jetrena pašteta, jetrena kobasica (40 g.)									
Pržena panirana riba (sa brašnom)									
Riblji štapići, panirana riba (brašno, jaja, krušne mrvice									
Bijela riba: orada, zubatac, brancin, bakalar									
Plava riba: losos, tuna, srdele, inćuni									
Plodovi mora: dagnje, škampi, rakovi									
Ikra									
**Kruh i pekarski proizvodi (veličina porcije -1 šnita ili 1 keks)**									
Bijeli kruh i pecivo									
Crni kruh i pecivo									
Integralni kruh i pecivo									
Krekeri (npr. Vic), pogačice od sira									
Suhi krekeri									

Prehrambene namirnice i količne **Prosječan unos namirnice unutar godine dana**

Žitarice (zdjela)	Nikad ili < od 1 mjesečno	1-3 puta mjesečno	1 tjedno	2-4 puta tjedno	5-6 puta tjedno	1 dnevno	2-3 puta dnevno	4-5 puta dnevno	6 ili > puta dnevno
Zobena kaša, palenta									
Cornflakes, muesli									
**Krumpir, riža i tjestenina (srednje velika porcija)**									
Kuhani krumpir, pire krumir, pečeni neoguljeni krumpir (180 g.)									
Pommes frites (100 g.)									
Pečeni krumpir u pečnici (180 g.)									
Krumpir salata (120 g.)									
Bijela riža (150 g.)									
Smeđa riža (150 g.)									
Tjestenina (bijela ili zelena) (230 g.)									
Integralna tjestenina (230 g.)									
Lazanje, musaka (400 g.)									
Pizza (240 g.)									
**Mliječni proizvodi i masti**									
Kiselo vrhnje (žlica)									
"Light" jogurt (125 g)									
Punomasni/grčki jogurt (125 g)									
Mliječni deserti (125 g)									
Sir, npr tilzit, gouda (40g.)									
Svježi sir (40g.)									
Jaja: kuhana, pečena.. (jedno)									
Quiche (120g.)									
Preljev za salatu sa smanjenim udjelom masti (15g.)									
Preljev za salatu na bazi majoneze (30g.)									
Francuski preljev za salatu (15g.)									
Ostali preljevi za salatu (15g.)									

**Prehrambene namirnice i količne**	**Prosječan unos namirnice unutar godine dana**								
Namazi	Nikad ili < od 1 mjesečno	1-3 puta mjesečno	1 tjedno	2-4 puta tjedno	5-6 puta tjedno	1 dnevno	2-3 puta dnevno	4-5 puta dnevno	6 ili > puta dnevno
Maslac (15g.)									
Margarin (15g.)									
Margarin sa polinezasičenim masnim kiselinama, npr Omegol (15g.)									
Margarin na bazi maslinovog ulja i mliječni namazi (15g.)									
Namazi sa manje od 60 % masti (15g.)									
Namazi sa manje od 30 % masti (15g.)									
Slastice i grickalice (veličina porcije -1 šnita ili 1 keks)									
Slatki keksi, čokoladni npr. digestive (jedan)									
Slatki keksi, jednostvani (petit beurre)									
Torta npr voćna, biskvit (domaća)									
Torta npr voćna, biskvit (kupovna)									
Slatka peciva, palačinke, krafne (domaća)									
Slatka peciva, palačinke, krafne, croasani (kupovna)									
Voćni kolači, pite, tartevi (domaći)									
Voćni kolači, pite, tartevi (kupovni)									
Puding, od mlijeka, riže (200g.)									
Sladoled (60 g)									
Čokolada (50 g)									
Čokoladice poput Marsa, Twixa…									
Bomboni (8 g)									
Dodani šećer npr u kavu, čaj.. (čajna žličica)									
Čips, smoki (40 g)									
Orašasti plodovi (20 g)									

Prehrambene namirnice i količne **Prosječan unos namirnice unutar godine dana**

Juhe, umaci i namazi	Nikad ili < od 1 mjesečno	1-3 puta mjesečno	1 tjedno	2-4 puta tjedno	5-6 puta tjedno	1 dnevno	2-3 puta dnevno	4-5 puta dnevno	6 ili > puta dnevno
Juha od povrća (220g.)									
Bistra juha od mesa (220g.)									
Bešamel umak, umak od sira, vrhnja, umak od pečenja (60g.)									
Kechup (30g.)									
Ajvar, pinđur (15g.)									
Džem, marmelada, med (15g.)									
Maslac od kikirikija (15g.)									
Pića									
Čaj (šalica)									
Kava (šalica)									
Kava bez kofeina (šalica)									
Kakao, vruća čokolada (šalica)									
Vino (125 ml)									
Pivo, jabukovača (250 ml)									
Slatka vina i slatka alkoholna pića (čaša)									
Žestoka alkoholna pića (čaša)									
Bezalkoholna pića niske kalorijske vrijednosti (čaša)									
Gazirana bezalkoholna pića (čaša)									
100% sok od naranče (čaša)									
100% sok od jabuke (čaša)									
Sok od sirupa za razrjeđivanje s vodom (čaša)									
Voće									
Jabuke (1 voćka)									
Kruške (1 voćka)									
Naranče, mandarine (1 voćka)									
Grejp (1 voćka)									

Banana (1 voćka)									

Prehrambene namirnice i količne	Prosječan unos namirnice unutar godine dana								
Voće (nastavak sa predhodne stranice)	Nikad ili < od 1 mjesečno	1-3 puta mjesečno	1 tjedno	2-4 puta tjedno	5-6 puta tjedno	1 dnevno	2-3 puta dnevno	4-5 puta dnevno	6 ili > puta dnevno
Grožđe (100 g)									
Dinja (1 šnjita)									
Breskva, šljiva, marelica (1 voćka)									
Jagoda, malina, kivi (80 g)									
Kompoti (100 g)									
Sušeno voće, grožđice, suha šljiva (30g)									
Povrće (svježe, smrznuto, konzervirano)									
Mrkva (60g.)									
Špinat (90g.)									
Brokula, kelj (90g.)									
Kelj pupčar (90g.)									
Kupus (90g.)									
Grašak (65g.)									
Mahune, bob (90g.)									
Tikvica, bundeva (224g.)									
Cvjetača (90g.)									
Pastrnjak, repa, koraba (70g.)									
Poriluk (160g.)									
Luk (90g.)									
Češnjak (10g.)									
Gljive (44g.)									
Paprika (160g.)									
Klice (60g.)									
Zelena salata, krastavac, celar (30g.)									
Potočarka (20g.)									
Rajčica (85g.)									
Kukuruz šećerac (60g.)									

Cikla (35g.)									
Salata od kupusa (45g.)									

**Prehrambene namirnice i količne**	**Prosječan unos namirnice unutar godine dana**								
Povrće (nastavak sa predhodne stranice)	Nikad ili < od 1 mjesečno	1-3 puta mjesečno	1 tjedno	2-4 puta tjedno	5-6 puta tjedno	1 dnevno	2-3 puta dnevno	4-5 puta dnevno	6 ili > puta dnevno
Avokado (145g.)									
Zapečeni grah (135g.)									
Sušena leća, grah, grašak (30g.)									
Tofu, soja, proteini povrća, veganski burger (60g.)									

Postoji li hrana koju ste jeli unutar godine dana a da nije obuhvaćena ovim upitnikom? Ako da, molimo naznačite u listi u nastavku

Vrsta hrane	Veličina serviranja	Broj konzumiranja unutar tjedan dana

Koju vrstu mlijeka najčešće koristite? Odaberite samo jedan odgovor

Punomasno mlijeko (3,5 % -4,0 % m.m.)		Poluobrano mlijeko (1,5%-2,8% m.m.)	
Obrano mlijeko (do 0,5 % m.m.)		Standardizirano ekstra punomasno mlijeko (4,0%-9,0% m.m)	
Mlijeko u prahu		Mlijeko od soje	
Ostala mlijeka		Niti jedno	

Koju ukupnu količinu mlijeka dnevno konzumirate (uključujući u kavi, čaju, sa žitaricama)?

Ne konzumiram mlijeko		350 ml	
120 ml		500 ml	
240 ml		više od 500 ml	

Da li uobičajeno za doručak jedete žitarice (navedeno pitanje se odnosi na kupovne gotove žitarice)?

Da		Ne	

Ako da, molim Vas navedite jedan ili dva primjera žitarica koje najčešće koristite i od kojeg su proizvođača.

Proizvođač	Ime žitarica

Koju vrstu masnoća najčešće koristite kod kuhanja? Molimo izaberite samo jedan odgovor.

Maslac		Margarin	
Mast		Ne koristim	
Biljno ulje			

Ako koristite kod kuhanja biljno ulje molim vas navedite koje

Koju vrstu masnoća najčešće koristie kod izrade kolača? Molimo izaberite samo jedan odgovor.

Maslac		Margarin	
Mast		Ne koristim	
Biljno ulje			

Ako kod izrade kolača koristite margarin, molim Vas navedite koji margarin koristite i od kojeg proizvođača

Koliko često jedete hranu koju pržite doma?

Svakodnevno		4-6 puta tjedno	

1-3 puta tjedno		Nikad	
Manje od jednom tjedno			

Koliko često jedete prženi hranu vani?

Svakodnevno		4-6 puta tjedno	
1-3 puta tjedno		Nikad	
Manje od jednom tjedno			

Što radite se vidljivom masnoćom na mesu?

Većinu pojedem		Pojedem što je manje moguće	
Pojedem manji dio		Ne jedem uopće to meso	

Koliko puta tjedno jedete pečeno meso?

Pečeno meso najčešće termički obradim do stupnja:

Izrazito pečenog mesa(tamno smeđe boje)	
Srednje pečenog mesa	
Lagano termički obrađenog (skoro sirovog)	
Ne jedem meso	

Koliko često dodajete sol u hranu za vrijeme kuhanja?

Uvijek		Rijetko	
Često		Nikad	
		Ponekad	

Koliko često dodajete sol u hranu nakon kuhanja (za vrijeme obroka)?

Uvijek		Rijetko	
Često		Nikad	
		Ponekad	

Da li redovito koristite zamjenu za sol (npr. Supisol)?
Ako da, koju?

Za vrijeme obroka, koliko ste prosječno unutar godine dana konzumirali slijedeću hranu?

		Broj obroka tjedno
Povrće (ne uključuje krumpire)		
Salate		
Voće i proizvodi od voća (ne uključuje voćne sokove)		
Ribu i riblje proizvode		
Mesom, mesna jela i mesne poizvode (uključuje slaninu, šunku i piletinu)		

Hvala Vam na pomoći
